# Efficient denoising in LED-based optoacoustic tomography with squeeze-and-excitation deep convolutional networks

**DOI:** 10.1117/1.JBO.31.4.046003

**Published:** 2026-03-31

**Authors:** Yuan Xu, Xiang Liu, Xosé Luis Deán-Ben, Sandeep Kumar Kalva, Daniel Razansky

**Affiliations:** a University of Zurich, Institute of Pharmacology and Toxicology and Institute for Biomedical Engineering, Faculty of Medicine, Zurich, Switzerland; b ETH Zurich, Institute for Biomedical Engineering, Department of Information Technology and Electrical Engineering, Zurich, Switzerland; c Indian Institute of Technology Bombay, Department of Biosciences and Bioengineering, Powai, Mumbai, India

**Keywords:** optoacoustic imaging, photoacoustics, deep learning, artifacts, denoising, light-emitting diode

## Abstract

**Significance:**

Low-cost optoacoustic imaging based on light-emitting diodes (LEDs) offers an affordable alternative to traditional laser-based systems, potentially broadening the reach of this technology into resource-limited settings. However, LEDs are only able to excite very weak optoacoustic responses, which leads to prominent noise artifacts in the reconstructed images.

**Aim:**

We aim to mitigate noise-related artifacts in LED-based optoacoustic tomography and thereby enhance the image quality and usability of these low-cost systems.

**Approach:**

We propose a squeeze-and-excitation U-Net-based model (SE-UNet) for noise artifact reduction. The network incorporates a VGG19 convolutional neural network mid-layer feature extractor as a loss evaluation module. It is trained on noisy data paired with high-quality reference images generated using a conventional solid-state pulsed laser source.

**Results:**

Our model achieves consistent improvements on no-reference image-quality metrics (NIQE and BRISQUE) and in the contrast-to-noise ratio, effectively reducing noise artifacts while preserving image structure and details. In addition, it exhibits a rapid processing time of ∼3  ms per 480×480  pixel image on a GTX 2070 GPU, utilizing 627 MB of memory.

**Conclusions:**

These results highlight the potential of the proposed SE-UNet model for optimizing the performance of LED-based optoacoustic imaging systems, offering both high efficiency and improved image quality.

## 1 Introduction

Most existing optoacoustic (OA) imaging systems rely on bulky, expensive solid-state lasers (SSL) for optical excitation.^1^^–^^3^ This poses a major barrier for disseminating the technology, particularly in middle- and low-income countries.^4^^–^^6^ To address this limitation, alternative pulsed light sources, such as light-emitting diodes (LEDs), are increasingly being adopted.^7^ LED-based systems offer a more compact and cost-effective solution compared with traditional SSL-based solutions.^8^^,^^9^ However, LEDs deliver low (sub-mJ) per pulse energies^10^^–^^12^ compared with traditional pulsed lasers (10 to 100 mJ),^13^^–^^16^ thus resulting in a compromised signal-to-noise ratio (SNR) that is directly proportional to the per-pulse energy.^17^ This increases the prominence of noise and various types of artifacts in the images.^18^

Several LED-based optoacoustic tomography (OAT) systems have been developed with different light delivery geometry and acoustic detection configurations. Commercially available systems are commonly based on stacked arrays of LEDs arranged on either side of a linear array transducer,^8^^,^^19^ a configuration that is known to produce limited-view artifacts and suboptimal image quality in tomographic applications.^20^ This can be overcome by rotating the linear array together with LED arrays around the target object, at the expense of low temporal resolution and other accompanying motion artifacts.^21^ Recently, full-view LED-based optoacoustic tomography (FLOAT) was developed. It employs a stacked array of LEDs integrated onto a circular array transducer.^7^ It features accurate tomographic imaging performance with a full 360-deg panoramic illumination and simultaneous acoustic detection. Nevertheless, the image quality is compromised by the low per-pulse energy and prominent electromagnetic interference (EMI) noise. Although noise refers to unstructured, stochastic disturbances (e.g., thermal or parasitic noise^22^), ring patterns are classified as artifacts.^18^ These patterns arise from parasitic noise peaks in the original sinogram, which propagate through reconstruction algorithms and manifest as structured rings in the final image.

Deep-learning-based denoising methods—including U-Net, SegNet, fully convolutional networks (FCNs), GANs, and Noise2Noise—have been widely explored in OA imaging, targeting both the raw signal and reconstructed image domains.^18^^,^^23^^–^^26^ Although these techniques have demonstrated notable performance in reducing EMI noise and improving visual quality under various settings,^27^^–^^29^ LED-based OAT systems introduce additional challenges due to their inherently lower SNR and reduced energy budget. In addition, resource-limited implementations imply the development of denoising models that are not only effective but also computationally lightweight and readily deployable in portable environments. Recent studies have reported good denoising performance with CNN/U-Net-style models (e.g., U-Net enhanced real-time imaging^23^) and with semi-synthetic RF-domain training to improve needle visibility in AcousticX systems,^30^ highlighting the promise of deep learning for LED-based OAT.

In this study, we propose a modified squeeze-and-excitation U-Net (SE-UNet), designed to suppress structured artifacts (e.g., ring artifacts) in the reconstructed images acquired using the FLOAT system. The model is trained on high-quality images of human finger joints and whole-body mouse images, which were recorded with SSLs and merged with noise patterns from FLOAT acquisitions. Denoising performance was evaluated on SSL-generated images using full-reference metrics, namely, structural similarity index (SSIM), peak signal-to-noise ratio (PSNR), and visual information fidelity (VIF). Performance with the LED-generated images was then evaluated using a no-reference naturalness image quality evaluator (NIQE) and a blind/referenceless image spatial quality evaluator (BRISQUE) metric. Comparisons with a representative diffusion-based denoiser and BM3D are provided. Low-contrast anatomical structures are shown to be preserved in addition to major organs and the surrounding vasculature. The proposed SE-UNet consistently outperformed baselines and achieved efficient inference on both GPUs and edge devices.

## 2 Methods

### 2.1 Full-View LED-Based Optoacoustic Tomography System (FLOAT)

The imaging system employs 16 stacked arrays of high-power LEDs (SFH4171S, OSRAM, Munich, Germany), with each array consisting of 10 LEDs. The arrays were arranged circularly around the imaged subject, emitting light pulses with a peak energy of ∼0.48  mJ per pulse. The generated OA waves were detected by a custom-made full-ring ultrasound transducer array (Imasonic Sas, Voray, France) consisting of 512 detection elements equally distributed on 2 arcs with 40 mm radius, each with 174 deg angular coverage. Each sensing element of the array operates at a central frequency of 5 MHz, offering a nominal detection bandwidth exceeding 60%. The array is cylindrically focused in the elevational direction at a focal distance of 38 mm.^7^

### 2.2 SSL-Based OAT System

The SSL-based system is described in detail elsewhere.^31^ Briefly, it employs an Nd:YAG-based optical parametric oscillator (OPO) laser operating at a wavelength tuned to 850 nm and delivers a per-pulse energy of 52.8 mJ. The laser energy density of $∼5.9\;\mathrm{mJ}/{\mathrm{cm}}^{2}$ was maintained on the surface of the imaged subject. The light was coupled into a custom-made fiber bundle bifurcating into 12 individual output ferrules arranged on top and bottom of the custom-made full-ring array transducer.

The OA signals detected in both FLOAT and SSL-based systems were digitized by a custom-designed parallel data acquisition (DAQ) system at a sampling rate of 40 mega samples per second. The data were then transferred to a PC via a 1  Gb/s Ethernet connection for storage and subsequent processing. Data acquisition is controlled using a PC-based MATLAB (R2020b) interface.

### 2.3 Data Acquisition and Image Reconstruction

#### 2.3.1 SSL-based data acquisition and reconstruction

**Fig. 1:**
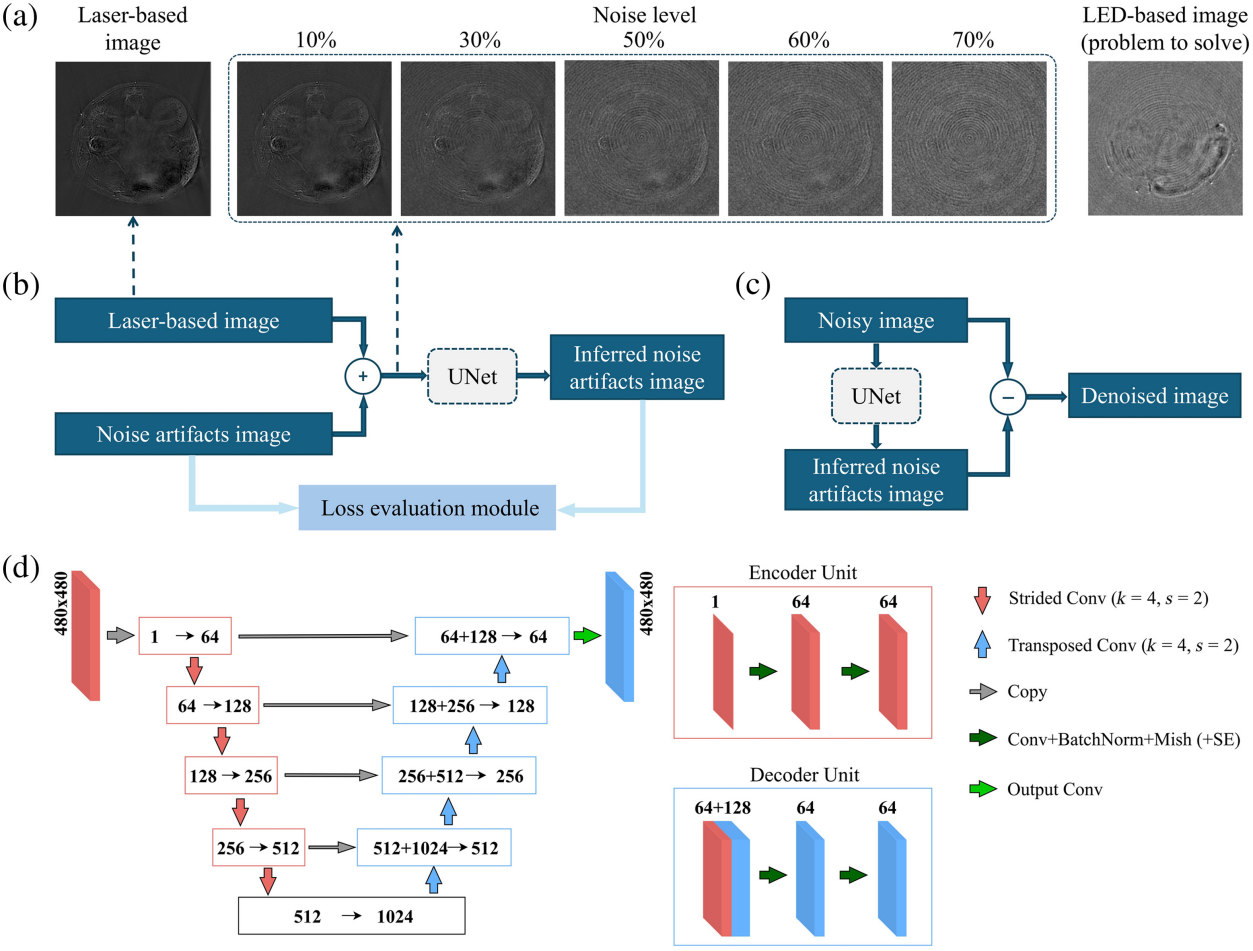
Image reconstruction and denoising pipeline. The proposed network was applied after the standard preprocessing/reconstruction steps (e.g., background subtraction followed by notch filtering and statistically weighted model-based reconstruction) and was intended to suppress residual artifacts rather than replace these established steps. (a) Left: Cross-sectional image of a mouse acquired with the SSL-based OA tomography system. Middle: equivalent mouse images after adding different levels of artificially generated noise artifacts. The noise level is defined by the pixel value weights of the combined images. For example, a 50% noise level implies the signal and noise artifacts have an equal weight. Right: The corresponding image acquired with FLOAT (after background noise subtraction). It can be observed that the FLOAT image approximates a 60% noise level. (b) Model training procedure: the high-quality SSL-based system image is combined with the noise artifacts image to create a noisy image, which is then fed into the model. The model infers the noise artifacts, and the loss evaluation module (whether a feature extractor or loss function) provides gradient descent information based on the difference between the inferred and actual noise artifacts. (c) Model testing procedure: a noisy image is input into the trained model, which infers the noise. The inferred noise is subtracted from the noisy image to obtain a denoised image. (d) SE-UNet architecture integrating SE blocks (channel-wise feature recalibration) and Mish activations, respectively. The diagram illustrates the data flow through convolutional layers, batch normalization, and Mish activations, with the encoder–decoder architecture enabling efficient feature extraction and reconstruction.

The index and middle fingers of both right and left hands of 11 healthy male and female volunteers were imaged using the SSL-based system. Different anatomical locations (5 to 7) of each finger were scanned with a 6 mm gap between them. In total, 255 finger cross-sections were scanned. At each cross-section, OA data for seven different wavelengths (700, 730, 760, 800, 850, 900, and 1064 nm) were sequentially acquired. In total, 650 cross-sectional finger images were reconstructed. A total of 1000 frames were averaged for each wavelength and at each cross-section. Similarly, 17 mice were scanned, and 650 cross-sectional images were reconstructed at different anatomical locations. Mice housing, handling, and experimentation were performed in accordance with the Swiss Federal Act on Animal Protection and were approved by the Cantonal Veterinary Office Zurich. An exemplary cross-sectional image of a mouse acquired by the SSL-based system is shown in Fig. 1(a). A model-based reconstruction technique was employed to obtain all the cross-sectional images.^32^

#### 2.3.2 FLOAT data acquisition and reconstruction

We employed the FLOAT imaging system to scan the mice at six different anatomical cross-sections, with a sampling rate of 50 Hz. We first utilized the previously reported signal and image processing pipeline to minimize background noise and optimize image quality.^7^^,^^33^ Briefly, the signal processing steps involved initially subtracting the background noise from the target object signal. Background noise was collected without any absorbers present in the field of view, immediately after the target object was scanned and removed from the imaged region. Furthermore, notch filtering and a statistically weighted model-based reconstruction technique were applied to obtain the cross-sectional images.^34^ Although the background is acquired without absorbers, the apparent artifacts in object-present reconstructions can differ due to the reconstruction method and the superposition of structured interference patterns on anatomy. We therefore treat the dominant artifacts in FLOAT as approximately additive in the reconstructed image domain and reduce mismatch by acquiring the background immediately after each object scan and by training with a diverse set of measured noise-only realizations. The denoising model was ultimately evaluated on human finger and mice images obtained with FLOAT, which implicitly reflects such effects. Each FLOAT image was obtained by averaging around 200 consecutive frames to suppress random noise. Notably, multiple background noise acquisitions demonstrated that the residual noise/artifacts were not exactly repeatable and can vary even after standard background subtraction [Fig. S1(a) in the Supplementary Material]. To further quantify this variability, we analyzed N=1315 background-only acquisitions and computed pairwise SSIM and PSNR across ≈20,000 acquisition pairs, yielding mean SSIM≈0.20 and mean PSNR≈17.5  dB with 5th to 95th percentile ranges of 0.16 to 0.23 and 15.5 to 19.2 dB, respectively (Fig. S2 in the Supplementary Material). Experimental tests indicated that averaging a larger number of frames did not yield notable improvements in image quality partly because motion-related artifacts become more pronounced when combining too many low-SNR frames. Although these processing steps facilitated noise reduction in sinograms, some significant parasitic noise persisted and propagated through the reconstruction algorithm.

#### 2.3.3 Motion correction

A pre-processing motion rejection algorithm was employed to avoid blurring due to respiratory motion, based on multiframe acquisition of time-resolved OA signals.^35^ An autocorrelation matrix of all frames was then computed. Clustering of frames was subsequently done by applying the second-order k-means method to the matrix of correlation coefficients. After rejecting frames afflicted with motion, the remaining frames were then averaged. Note that the motion-rejection algorithm was employed as the first signal processing step before subtracting the background noise. For FLOAT acquisitions, this procedure was not applied because the single-frame SNR was insufficient for reliable motion assessment.

#### 2.3.4 Data augmentation

Given the diverse noise artifact patterns and the need for robust model performance, we implemented various data augmentation techniques to enhance dataset diversity and model generalization. Data augmentation is particularly crucial in scenarios where large image datasets are unavailable. In this experiment, we expanded our dataset, which originally consisted of 550 mouse images and 550 finger images, by 10-fold using these techniques.

Specifically, basic techniques, such as cropping, scaling, rotating, flipping, and brightness/contrast adjustments, were utilized. In addition, we applied slight elastic deformations to the finger and mouse images obtained with the SSL-based system. This is permissible because the model’s task is to infer noise artifacts in the images. For the noise artifact images, data augmentation incorporated the aforementioned basic techniques, as well as the addition of white noise and salt-and-pepper noise. These augmentations were designed to mitigate overfitting and improve the model’s capacity to generalize to unseen data. The number of experiments and corresponding images are shown in Table 1.

**Table 1:** Categories, size, and relationship of the dataset.

Device	Description	Train set	Val. set	Test set
SSL-based system	Noise-free mouse slices	550 (5500 after augmentation)	70	30
	Noise-free finger slices	550 (5500 after augmentation)	70	30
Low-cost FLOAT system	Pure noise artifacts	1100 (11,000 after augmentation)	140	60
	Noisy mouse slices	\	\	5
	Noisy finger slices	\	\	3

### 2.4 Training Model and Testing Procedure

To create the training dataset, we combined images with pure noise artifacts obtained from the FLOAT system with SSL-based reconstructed images. This was done to replicate the noisy images generally rendered with FLOAT [Fig. 1(a)]. Specifically, an ample number of images with pure noise artifacts were reconstructed and denoted as $I_{\text{noise}}$, whereas the signal images were labeled as $I_{\text{signal}}$. The synthetic noisy images $I_{\mathrm{syn}-\text{noisy}}$ were then generated by pixel-wise weighted addition of the noise and signal images in the image domain according to the formula: $I_{\mathrm{syn}-\text{noisy}}=w*I_{\text{noise}}+(1-w)*I_{\text{signal}}$, where w denotes the noise level. Although this linear mixing does not fully capture all FLOAT-specific systematics, it provides a reproducible FLOAT-noise-aware training signal without requiring access to raw data. More importantly, this strategy enables the generation of paired noisy/clean images with well-defined ground truth, which would be unfeasible to obtain directly from real FLOAT acquisitions. The resulting images were then fed into the network, which was tasked with inferring the noise artifacts. This approach was employed during the training process [Fig. 1(b)]. The proposed model was trained to infer the noise artifacts within the images rather than the target object features. We predict the residual noise/artifacts (rather than the clean image) because these degradations are typically more stationary than the anatomy and thus easier to learn. Then, the final output was obtained by residual subtraction, which provides a conservative correction and mitigates over-smoothing/hallucination in low-SNR regions. The inferred noise artifact image I^noise was then compared with the $I_{\text{noise}}$ and the loss was computed to iteratively update the network parameters. To test the trained network [Fig. 1(c)], two types of images were used as noisy inputs $I_{\text{noisy}}$. The first type consisted of synthetic noisy test images composed of signals and noise artifacts entirely unseen during training. The second type consisted of FLOAT images with intrinsic noise artifacts. The inferred noise I^noise was then subtracted from the noisy images to restore their quality. This operation can be expressed as Irestored=Inoisy−I^noise. Training a model on an RTX 2070 takes ∼2 days.

### 2.5 Deep Learning Models

To identify the optimal loss evaluation module, we initially employed the BatchNorm UNet, training it exclusively on the original 550 finger images instead of the SE-UNet. The BatchNorm UNet was chosen for its greater simplicity, minimizing the risk of overfitting, especially with the small dataset of 550 finger images. This strategy reduced the impact of overfitting on the selection of the loss evaluation module. For the final denoising task, we then applied the SE-UNet using the best loss evaluation module identified.

1. BatchNorm UNet: The architecture is based on Dehner’s work.^18^ This network follows the classic UNet structure^36^ with added batch normalization layers after each convolutional layer. Leaky ReLU activation was used instead of the standard ReLU. These modifications stabilize the learning process, accelerate convergence, and prevent issues such as the dying ReLU problem.
2. SE-UNet: We augmented the U-Net with SE blocks that perform channel-wise recalibration using global context.^37^^,^^38^ In all the reconstructions, this introduced a bias toward suppressing globally coherent ring-like artifacts, with a possible mild smoothing trade-off. Mish was used as the stable nonlinearity activation function rather than a guaranteed performance booster.
3. Diffusion model: For baseline comparison, we implemented a diffusion-based denoiser following the improved standard denoising diffusion probabilistic model (DDPM) paradigm.^39^^,^^40^ Gaussian noise is progressively added to clean SSL-based images in the forward process and removed via learned reverse sampling. The model was trained on the same SSL-based dataset employed for SE–UNet and does not incorporate FLOAT-specific noise priors. Owing to its iterative sampling procedure, the diffusion model incurs a considerably longer inference time (∼240  ms per 480×480 image), but it serves as the basic deep-learning-based reference in our experiments.

### 2.6 Assessment of the Loss Evaluation Module and Denoising

We conducted three different experiments to identify the best loss evaluation module and evaluate the model’s performance on real experimental datasets.

#### 2.6.1. Experiment 1: identifying the optimal loss evaluation module for noise artifact removal

The goal of this experiment was to identify the most suitable loss evaluation module for removing noise artifacts. Given the inherent complexity of the SE-UNet model, there is a potential risk of overfitting on small datasets, which could compromise the assessment of the loss evaluation module. To address this, a simpler BatchNorm UNet model was employed for comparative experiments and trained exclusively on finger images. A variety of loss evaluation modules were compared, including network-based feature extractors derived from intermediate layers of pre-trained models (VGG19, VGG16, ResNet50),^41^^–^^43^ as well as mathematically defined losses such as mean squared error (MSE), mean absolute error (MAE), structural similarity index (SSIM), and multiscale structural similarity index (MS-SSIM).^44^^,^^45^ The best-performing loss evaluation module from this experiment was utilized in the subsequent experiments.

#### 2.6.2. Experiment 2: evaluating denoising performance on SSL-based images

In this experiment, the SE-UNet model was trained on mouse and finger images acquired using an SSL-based system, along with pure noise artifact images acquired using the FLOAT system, as illustrated in Fig. 1(b). To enhance the diversity of the training data, the dataset was augmented 10-fold, yielding 5500 images each for the mouse and finger categories, and 11,000 images of pure noise artifacts, as summarized in Table 1. Data augmentation techniques were applied only to the training set, whereas the validation and test sets remained unaltered. It is important to note that the training, validation, and test datasets are mutually exclusive to prevent data leakage. The model was trained using the optimal loss evaluation module identified in experiment 1 and tested on the SSL-based image test set.

#### 2.6.3. Experiment 3: generalization assessment on FLOAT images

Next, the trained model was evaluated on the noisy FLOAT images, where the noise artifacts were naturally present in the images, rather than being artificially introduced, following the workflow outlined in Fig. 1(c). This experiment assesses generalization under the practical constraints of FLOAT, where the combination of low SNR, structured artifacts, and limited availability of paired ground truth complicates direct benchmarking of modern deep-learning denoisers. To enable a fair comparison with a contemporary deep-learning denoiser, we included a diffusion-based denoiser trained on the same SSL-based images under a standard Gaussian corruption schedule (see Sec. 2.5.3). This denoiser serves as our primary reference baseline for FLOAT experiments. We additionally report results from BM3D,^46^^,^^47^ a widely used classical filtering method, for historical reference. BM3D’s parameters (e.g., the PSD threshold) were tuned via grid search (optimal PSD = 0.3).

### 2.7 Method for Assessing Loss Evaluation Module

In medical imaging, accurate assessment of image quality is crucial for effective diagnosis and treatment planning. VIF is an advanced metric designed to assess image quality by evaluating how well visual information is preserved between a reference image and a processed image. The VIF metric leverages concepts from natural scene statistics, information theory, and the characteristics of the human visual system (HVS) to provide a comprehensive measure that aligns closely with human perception of image quality.^48^^,^^49^

The VIF score, calculated as the ratio of the mutual information between the reference image [ground truth (GT)] and the processed image (P) across all scales, is given by

$$\mathrm{VIF}=\frac{\sum_{s}\sum \;\log_{10}(1+\frac{g^{2}\cdot \sigma_{GT}^{2}}{\sigma_{v}^{2}+\sigma_{n}^{2}})}{\sum_{s}\sum \;\log_{10}(1+\frac{\sigma_{GT}^{2}}{\sigma_{n}^{2}})}.$$

Here, VIF works by decomposing two images into multiple scales and computing local statistics, such as variance ($\sigma^{2}$) and covariance ($\sigma_{GT\cdot P}$). The gain factor $g=\frac{\sigma_{GT\cdot P}}{\sigma_{GT}^{2}}$ represents the relationship between *GT* and P, whereas the noise variance $\sigma_{v}^{2}=\sigma_{P}^{2}-g\cdot \sigma_{GT\cdot P}$ quantifies the noise introduced during processing, and $\sigma_{n}^{2}$ is a fixed constant representing the assumed visual noise variance, based on human visual system sensitivity. The inner summation aggregates contributions from the local image regions for each scale s, whereas the outer summation combines the results across all scales. This score reflects how well the processed image retains the visual fidelity of the original image, with higher VIF values indicating better preservation of visual information.^48^ Note that VIF was used for SSL-based experiments with available references; for FLOAT images without references, we report NIQE/BRISQUE as reproducible no-reference indicators.

## 3 Results

The proposed method demonstrated consistent effectiveness in removing noise artifacts, leading to improved image quality for both FLOAT images and artificially corrupted SSL-based images. Specifically, the evaluation of FLOAT images (n=10) manifested notable enhancements in contrast-to-noise ratio (CNR), NIQE, and BRISQUE metrics. To comprehensively evaluate the effectiveness of our proposed denoising method, we thus conducted studies to: identify the optimal loss evaluation module, evaluate performance on SSL-based data, and assess generalization on FLOAT data. Note that the term “noise level” refers to the “noise artifacts level,” encompassing both random and structured components.

### 3.1 Evaluation of Optimal Loss Evaluation Module for Noise Artifacts Removal

**Fig. 2:**
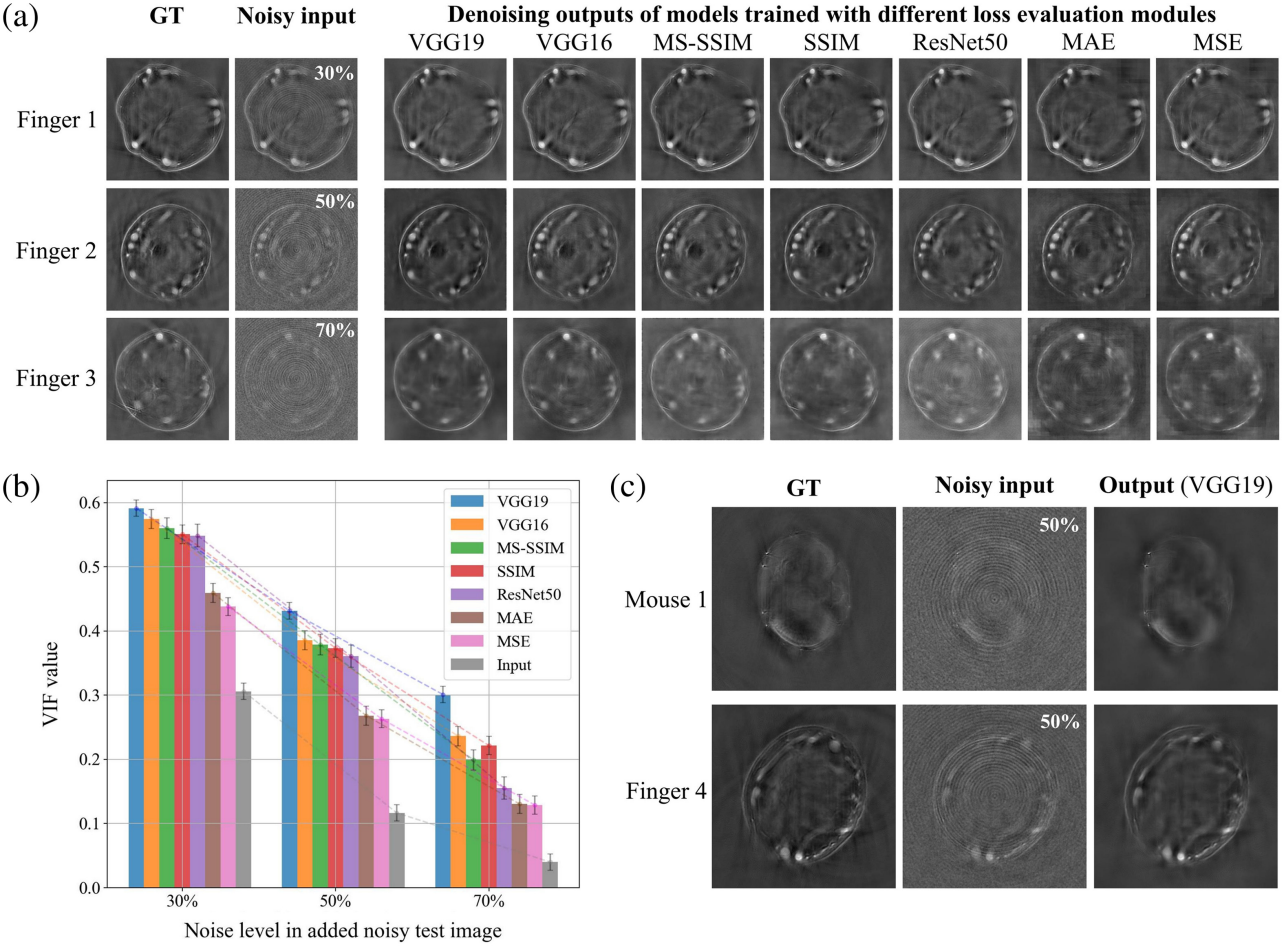
Impact of different loss evaluation modules on the model performance and effectiveness of image denoising. (a) Output images of models trained with various loss evaluation modules. All models were trained using the BatchNorm UNet on a dataset of 550 finger images with varying levels of artificially added noise artifacts, ranging from 10% to 90%, with identical training parameters except for the loss evaluation modules, which included VGG19, VGG16, MS-SSIM, SSIM, ResNet50, MAE, and MSE. All models were tested on 30 SSL-based finger images with artificially added noise levels set at 30%, 50%, and 70%. (b) Evaluation using VIF on the test set of 30 finger images, comparing the denoising results with the ground truth (GT). Higher VIF scores indicate closer resemblance to the GT. (c) Example results from the SE-UNet model trained with the VGG19-based loss metric on the augmented dataset comprising finger and mouse images, evaluated on the SSL-based test set. The left column shows the GT images, the middle column displays the input images with artificial noise artifacts added, and the right column illustrates the denoised output.

To determine the optimal loss evaluation module, training and testing were conducted on a dataset of finger joint images. The results, as shown in Fig. 2(a), include three high-quality SSL-based finger images, their counterparts as model input with noise artifacts added at 30%, 50%, and 70% levels, and the corresponding output quality produced by each model. Although the noise levels used for testing range from 30% to 70%, the training phase involved a broader range of 10% to 90%. This extended range was employed to enhance the model’s generalization capabilities. However, for validation and testing, we focused on the 30% to 70% noise range, which is more representative of the noise levels typically encountered with the FLOAT system.

The data presented in Fig. 2(a) reveal that models trained with MAE and MSE losses exhibit significant structural inconsistencies and blocky artifacts, likely due to their emphasis on pixel-wise accuracy at the expense of overall image structure. By contrast, models trained using MS-SSIM and SSIM losses, which consider overall structural similarity, retain better structural coherence, although some ring artifacts and distortions persist. The loss evaluation modules based on the VGG19, VGG16, and ResNet50 feature extractors leverage intermediate layers of pre-trained models to perform perceptual image quality assessment. Among them, VGG19 achieved the highest noise artifact removal efficiency and image fidelity. The superior performance of VGG19 can be attributed to its deeper architecture, which captures more detailed and representative image features. This allows the loss evaluation module to better preserve the global structure and fine textures.

The VIF bar chart in Fig. 2(b) offers a comprehensive evaluation of the denoising performance across different loss functions under varying noise levels. As noise increases, a noticeable decline in VIF scores across all models occurs, indicating the challenges posed by higher noise levels in retaining image fidelity. Notably, the VGG19-based model consistently achieves the highest VIF scores across all noise levels and visually balances effective denoising with detail preservation. At the 30% noise level, the VGG19 model yields a VIF score of ∼0.59, outperforming other models such as VGG16, MS-SSIM, and SSIM, which yet perform relatively well. As noise intensity escalates to 50% and 70%, the VIF scores for all models decrease, but VGG19 maintains a leading position with scores of around 0.43 and 0.30, respectively. This performance mismatch is especially pronounced for models trained with MSE and MAE loss functions, which show a consistent drop in VIF scores, particularly at higher noise levels (around 0.13 at 70% noise). The decline in performance for MSE and MAE suggests that these pixel-wise loss functions struggle to overcome high noise levels, arguably oversmoothing the images and losing critical details. Overall, VGG19 proves to be the most robust choice of the loss evaluation module for guiding UNet’s gradient descent, especially in high-noise scenarios.

### 3.2 Performance on SSL-Based Images with Artificially Added Noise Artifacts

In Fig. 2(c), the denoising performance of the model trained exclusively using the VGG19 loss evaluation module is presented. This model was trained on an augmented dataset that included both finger and mouse cross-sectional images in equal proportions. The images demonstrate the model’s ability to effectively remove the noise at the 50% noise level while preserving major anatomical features in both mouse and finger images.

Compared with previous images [Fig. 2(a)], where the model was trained solely on finger images, the inclusion of heterogeneous mouse data further validates the model’s robustness and broad applicability. The consistent denoising performance across both species indicates that the VGG19-based model is well-suited for diverse medical imaging tasks.

As an additional deep-learning-based baseline, we evaluated a diffusion-based denoiser trained on the same clean (SSL) images following a diffusion training protocol.^40^ On the 60-image test set, our proposed method outperformed diffusion in global PSNR, SSIM, and VIF metrics (details are presented in Table S1 and Figs. S3–S4 in the Supplementary Material), and achieved substantially faster inference (3 ms versus 240 ms) due to its efficient architecture.

### 3.3 Denoising Performance on FLOAT Images

**Fig. 3:**
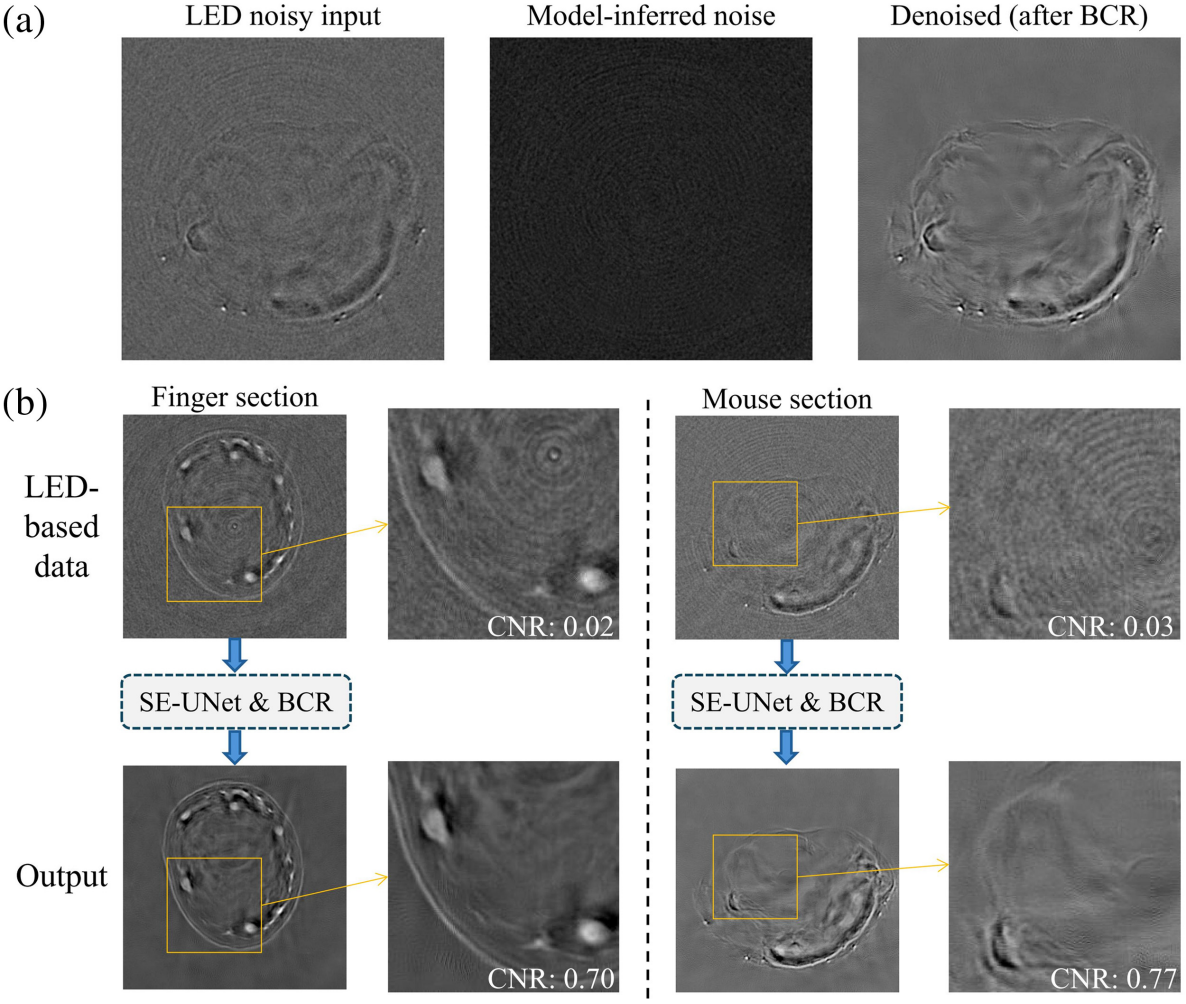
Denoising results on LED-based images. (a) Artifactual LED-based images after background noise subtraction are used as input. The model infers the noise artifact components in the image. The denoised image is obtained by directly subtracting the inferred noise artifacts from the original noisy image. These denoised images are typically very dark because the subtraction removes noise artifacts from pixel values, leaving only small signal values. To facilitate visual inspection, we applied global brightness and contrast restoration (BCR). (b) The top image shows the LED-based images with noise artifacts (after background noise subtraction). The bottom image displays the denoised results. For calculating CNR, the yellow rectangle represents the region of interest (ROI), whereas the background region is selected outside the finger/mouse area.

We next evaluated the denoising performance of the SE-UNet model on the FLOAT images of both finger and mouse cross-sections, which is the focus of this study. The primary goal was to address persistent noise artifacts, which are common challenges in LED-based systems. As illustrated in Fig. 3(a), the denoising process begins with noisy input images, where the model identifies and subtracts noise artifact components to produce denoised outputs. Brightness and contrast restoration (BCR) was performed to compensate for the loss in image quality caused by the subtraction process.

A detailed comparison [Fig. 3(b)] highlights the improvements in image quality before and after denoising. The CNR increased from 0.02 to 0.70 in finger images and from 0.03 to 0.77 in mouse images, indicating enhanced visibility of anatomical features. To provide a broader assessment under the realistic noise regime in FLOAT with ground truth unavailable, we further evaluated a supplementary set of 10 FLOAT images (three finger and seven mouse cross-sections) using NIQE and BRISQUE, two widely adopted no-reference image quality metrics.^50^^,^^51^ As summarized in Table 2, the proposed SE-UNet achieved the strongest overall improvement, reducing NIQE from 10.14±1.05 (noisy input) to 3.98±0.59, and BRISQUE from 35.48±2.38 to 20.89±7.55.

**Table 2:** Image quality scores (mean ± std) for 10 noisy versus denoised images (lower scores indicate better quality).

Metric	Noisy input	BM3D	Diffusion	Proposed
NIQE (↓)	10.14 ± 1.05	6.55 ± 0.17	7.61 ± 1.01	**3.98** ± 0.59
BRISQUE (↓)	35.48 ± 2.38	44.51 ± 1.04	29.91 ± 3.50	**20.89** ± 7.55

Note: bold values indicate the lowest (best) score among the compared methods for each metric.

The diffusion baseline, representing a state-of-the-art deep-learning denoiser, significantly improves image quality compared with the noisy input. The comparative results between diffusion and BM3D differ across BRISQUE and NIQE metrics. (NIQE: 7.61±1.01 versus 6.55±0.17; BRISQUE: 29.91±3.50 versus 44.51±1.04.) However, diffusion still falls short of the proposed SE–UNet, particularly in NIQE (3.98±0.59) and BRISQUE (20.89±7.55), likely due to the distribution difference between Gaussian training noise and structured FLOAT artifacts. By contrast, the proposed residual-subtraction formulation is explicitly tuned to remove measured artifacts and therefore achieves the best scores. BM3D, included for completeness, manifests inferior performance on several metrics.

In terms of efficiency, our method performs denoising in a single forward pass (∼3  ms) on a GTX 2070 GPU, supporting practical deployment in resource-constrained LED-based imaging systems (diffusion: ∼240  ms; BM3D: ∼2.4  s). On an NVIDIA Jetson Orin Nano edge device, the proposed model runs in ∼16  ms per frame (see Sec. 4 and Table S2 in the Supplementary Material for details).

## 4 Discussion and Conclusions

This study presents an SE-UNet model optimized with VGG19 features for denoising OA images acquired using low-cost LED-based systems. The model operates directly in the image domain, avoiding raw signal processing and enabling straightforward integration into image-based analysis pipelines. Experimental results indicate that using VGG19 as the loss evaluation module improves the denoising performance and preservation of structural details compared with other feature extractors (e.g., ResNet50) and conventional loss metrics (MSE, MAE, SSIM, and MS-SSIM). In addition, incorporating SE blocks into the U-Net introduced channel-wise feature recalibration with negligible computational overhead. In the FLOAT setting, ring-like patterns represent structured and globally coherent artifacts; SE blocks therefore bias the network toward stronger suppression of such artifacts, which can include a mild smoothing trade-off in low-contrast regions. Consistent with this interpretation, a paired SE ablation conducted under identical settings (trained on the original, nonaugmented dataset) showed a slight increase in PSNR (mean +0.19  dB) accompanied by marginal decreases in SSIM (∼−0.006) and VIF (∼−0.013) (Figs. S5–S6 in the Supplementary Material).

A major concern in denoising low-SNR images generated with FLOAT was the attenuation of weak, low-contrast anatomical structures together with background artifacts, especially when the denoiser exhibited mild smoothing. To explicitly quantify structural preservation beyond global averages, we reported: (i) global full-reference metrics (SSIM, PSNR, and VIF) on a 60-image SSL test set under the representative artifact setting used throughout this work (Table S1 in the Supplementary Material); (ii) ROI-based SSIM analyses focusing on low-contrast regions (Figs. S3–S4 in the Supplementary Material); and (iii) qualitative visualization of key structures in representative ROIs (Fig. S7 in the Supplementary Material). These localized evaluations are important because global metrics can mask subtle, but clinically relevant, image degradation concentrated in small structures. In this context, our residual-learning formulation—predicting artifact components and subtracting them from the input—acts as a conservative correction mechanism that reduces the incentive to hallucinate structures, and empirically yields stronger ROI structural fidelity under the tested artifact setting. To qualitatively assess preservation of clinically important anatomy, Fig. S7 in the Supplementary Material displays representative ROIs on mouse slices highlighting the spinal cord (ROI 2), major blood vessels such as the inferior vena cava and abdominal aorta adjacent to the spine (ROI 1), and smaller peripheral vessels (ROI 3). Compared with the noisy input and the diffusion baseline, the proposed method preserved the morphology and boundaries of these structures while suppressing ring artifacts and background noise. A diffusion-based denoiser trained under an improved Gaussian diffusion paradigm on SSL images is included in Table S1 in the Supplementary Material. This baseline does not explicitly model the measured background noise distributions in FLOAT; it is included to benchmark against a representative modern denoiser rather than to claim universal dominance.

Complementing these full-reference and ROI-based assessments, improvements in image quality were consistently observed, as reflected in lower NIQE and BRISQUE scores together with clearer visual appearance. Although these metrics were originally developed for natural images, we report them here as reproducible no-reference indicators, complemented by ROI-level CNR analysis and visual inspection to capture clinically relevant structures. These results suggest that the method effectively reduces noise artifacts while preserving essential image structures. The model achieves a processing time of ∼3  ms per frame on a GTX 2070 GPU, using 627 MB of memory. To assess edge-device deployability, we benchmarked inference latency on an NVIDIA Jetson Orin Nano using TensorRT FP16 (fixed input shape 480×480, batch size = 1) and obtained an end-to-end latency of ∼16  ms per frame under the 15 W nvpmodel setting (Table S2 in the Supplementary Material). This level of computational efficiency may support integration into systems with limited hardware resources, such as compact or portable OA devices. The computational efficiency of the proposed approach suggests its potential applicability to LED-based OAT systems operating at higher imaging frame rates, including those in the kilohertz range.

The proposed network may also be applicable to hand-held transducer arrays with reduced tomographic coverage, offering potential for increased portability in clinical imaging workflows. Furthermore, the fast processing time may support multispectral imaging scenarios.^52^^,^^53^ The training incorporated SSL-based images spanning 700 to 1064 nm, which may support wavelength-robust features, although formal testing of spectral-signature preservation and unmixing performance is left for future work. The developed network architecture is suitable for other OA imaging systems that employ high-repetition sources, such as laser diodes, where low per-pulse energy levels are often dictated by the need to conform to laser exposure limits and avoid tissue damage. We note that aggressive denoising can potentially attenuate low-contrast anatomical signals. Therefore, we provide ROI-based quantitative analyses and visual assessment to specifically examine structural preservation, and additional benchmarking against a representative diffusion-based denoiser and global quantitative results over 60 test images are provided in Table S1 and Figs. S3–S4 in the Supplementary Material.

In summary, we presented an optimized U-Net architecture with SE blocks and a VGG19-based perceptual loss for denoising reconstructed images in LED-based OA systems. The model demonstrated consistent noise reduction in both finger and mouse datasets while preserving relevant anatomical features. These findings indicate the method’s potential utility in cost-sensitive clinical and preclinical imaging applications.

## Supplementary Material

10.1117/1.JBO.31.4.046003.s01

## Data Availability

The datasets and code used during the current study are available from the corresponding author upon reasonable request.
