# Supplementary material for: Efficient denoising in LED-based optoacoustic tomography with squeeze-and-excitation deep convolutional networks
Source: J Biomed Opt. 2026 Mar 31;31(4):046003. doi: 10.1117/1.JBO.31.4.046003 (PMC13037428; doi:10.1117/1.JBO.31.4.046003)
Supplement: Supplementary file 1 [file JBO_031_046003_SD001.pdf]

SUPPLEMENTARY FIGURES AND TABLES

Supplementary Fig. S1. Variability of background measurements across acquisitions and the role of background subtraction in the sinogram-domain pipeline.

Supplementary Fig. S2. Quantitative characterization of inter-acquisition background variability in FLOAT.

Supplementary Fig. S3. Quantitative and ROI-based evaluation on a finger slice.

Supplementary Fig. S4. Quantitative and ROI-based evaluation on a mouse slice.

Supplementary Fig. S5. SE-block ablation (paired N=60): U-Net w/ SE vs U-Net w/o SE; Δ = (U-Net w/ SE) − (U-Net w/o SE).

Supplementary Fig. S6. Representative qualitative ablation results on SSL-based samples

Supplementary Fig. S7. Visualization of Key Clinical Structures in Representative ROIs.

Supplementary Table S1. Global quantitative evaluation over 60 SSL-based test images (mean ± std).

Supplementary Table S2. Embedded inference benchmark on Jetson Orin Nano.

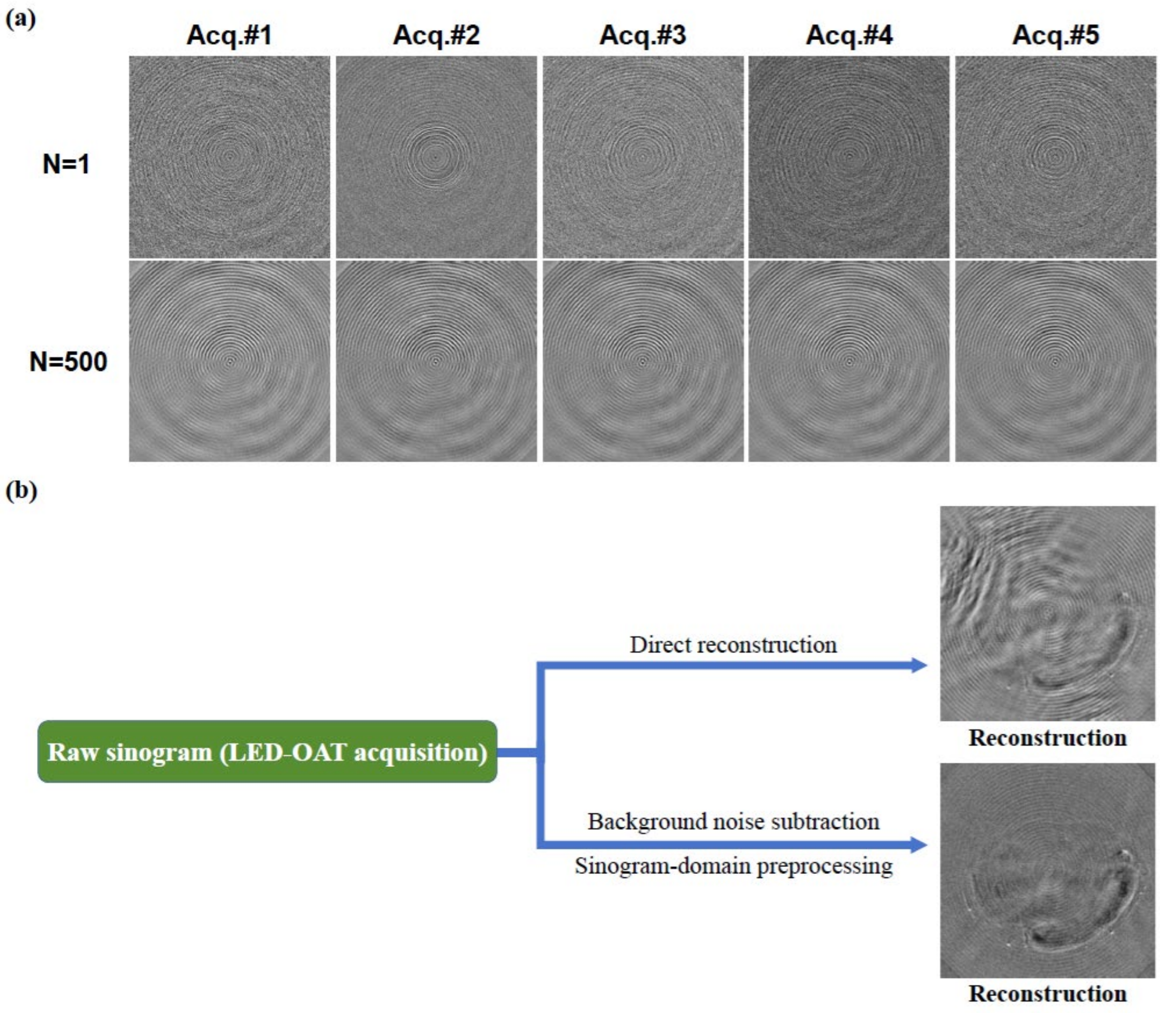

(a) Background-only reconstructions from five independent acquisitions (no absorber in the field of view) are shown for N=1 and N=500 frame averaging (N=500 ≈ 10 s). Single-frame backgrounds exhibit strong realization-to-realization variability, whereas averaging suppresses stochastic noise and reveals a similar ring-like pattern. All panels are displayed with identical intensity range in the image domain; nevertheless, the underlying sinogram-domain ring component remains non-repeatable, showing substantial value differences and pointwise fluctuations within and across sessions. (b) Processing overview and an example reconstruction with vs. without background subtraction. After each subject acquisition, a background-only measurement is acquired immediately (subject removed) to capture contemporaneous background. Background subtraction and preprocessing (frame averaging, notch filtering, motion correction, etc.) are performed in the raw sinogram domain prior to reconstruction. Although these steps markedly reduce artifacts, residual ring-dominated interference can persist, motivating additional post-processing.

Supplementary Fig. S1

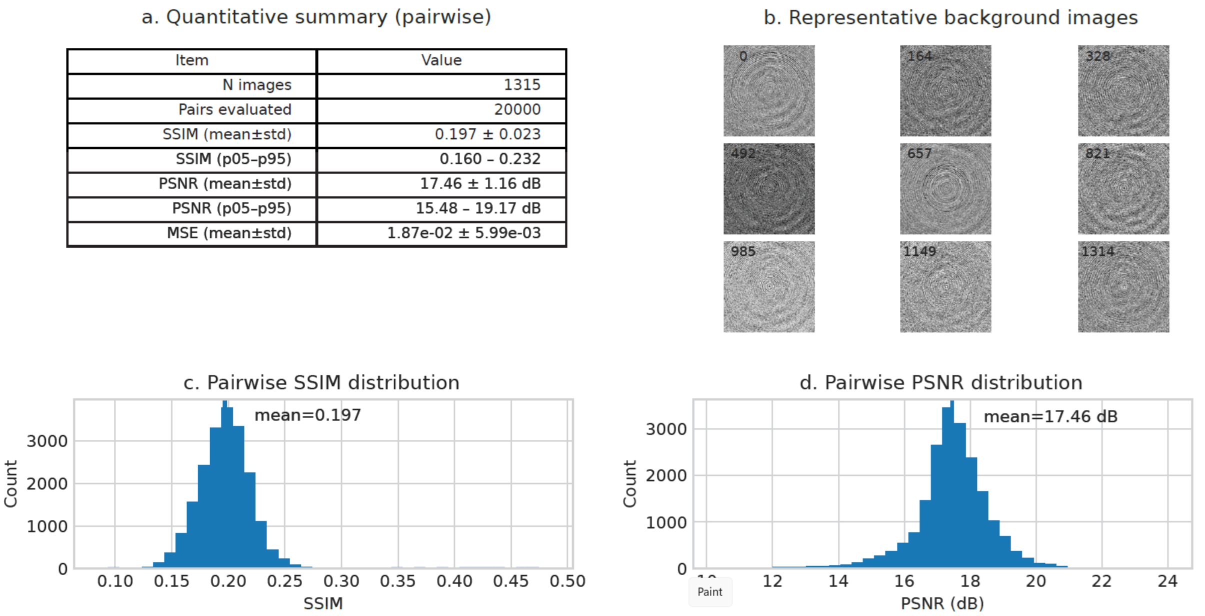

Item	Value
N images	1315
Pairs evaluated	20000
SSIM (mean±std)	0.197 ± 0.023
SSIM (p05–p95)	0.160 – 0.232
PSNR (mean±std)	17.46 ± 1.16 dB
PSNR (p05–p95)	15.48 – 19.17 dB
MSE (mean±std)	1.87e-02 ± 5.99e-03

Background-only reconstructions (no absorber in the field of view) were analyzed across N = 1315 acquisitions. (a) Quantitative summary of pairwise comparisons (20,000 acquisition pairs), reporting SSIM, PSNR, and MSE (mean ± std and 5th–95th percentiles). (b) Representative background reconstructions displayed with a consistent intensity range (global 1st/99th percentiles) to avoid per-panel auto-contrast. (c) Histogram of pairwise SSIM values across acquisition pairs (mean 0.197). (d) Histogram of pairwise PSNR values across acquisition pairs (mean 17.46 dB). Together, these statistics indicate pronounced interacquisitionvariability, supporting that standard background subtraction can reduce artifacts but may leave acquisitiondependentresiduals.

Supplementary Fig. S2

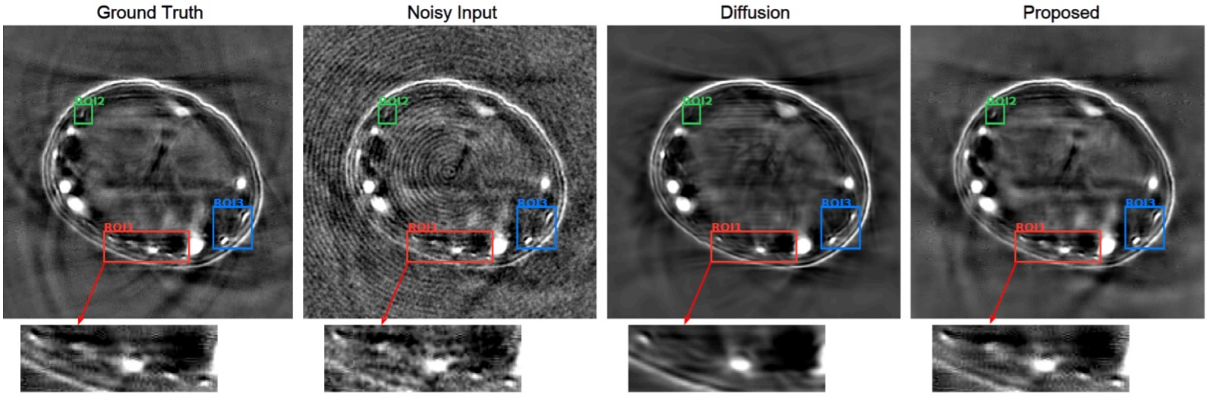

1. Global metrics (vs Ground Truth)

Metric	Noisy Input	Diffusion	Proposed
SSIM	0.4312	0.5659	**0.8257**
PSNR	20.25	21.46	**27.16**
VIF	0.2011	0.1640	**0.3397**

2. ROI SSIM (vs Ground Truth)

ROI	Noisy Input	Diffusion	Proposed
1	0.7432	0.6058	**0.8729**
2	0.6602	0.4494	**0.8175**
3	0.7483	0.5184	**0.8470**

Supplementary Fig. S3

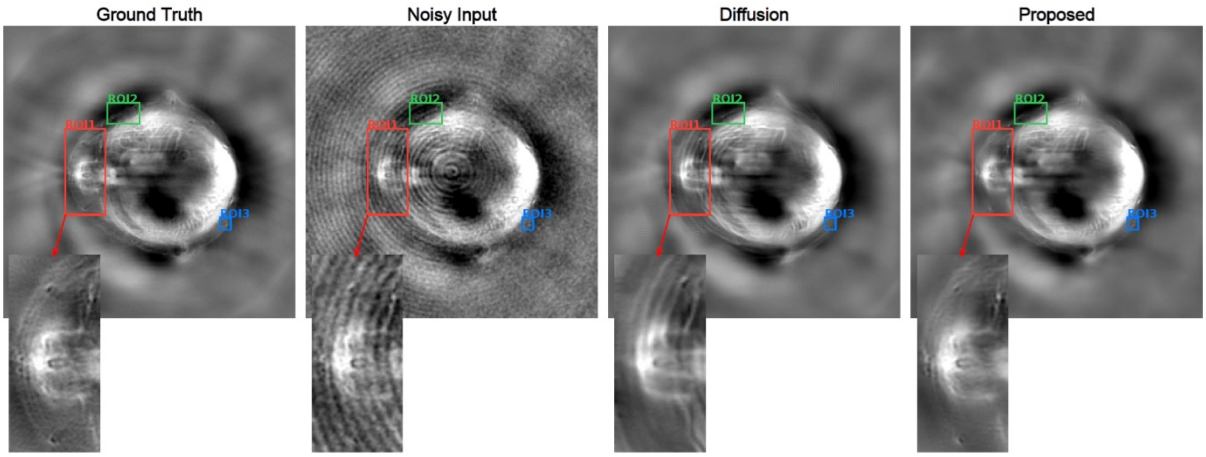

1. Global metrics (vs Ground Truth)

Metric	Noisy Input	Diffusion	Proposed
SSIM	0.4853	0.8106	**0.9155**
PSNR	25.11	29.42	**30.31**
VIF	0.2726	0.3811	**0.5189**

2. ROI SSIM (vs Ground Truth)

ROI	Noisy Input	Diffusion	Proposed
1	0.5441	0.6632	**0.8658**
2	0.6729	0.7566	**0.8946**
3	0.7894	0.6481	**0.8678**

Supplementary Fig. S4

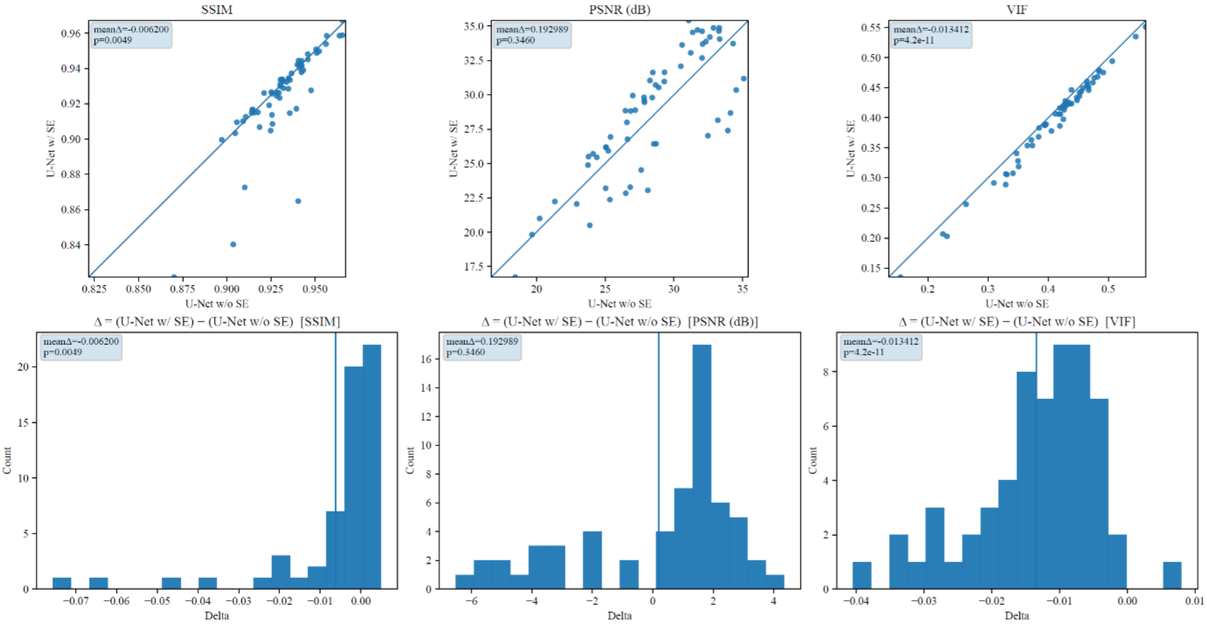

Supplementary Fig. S5

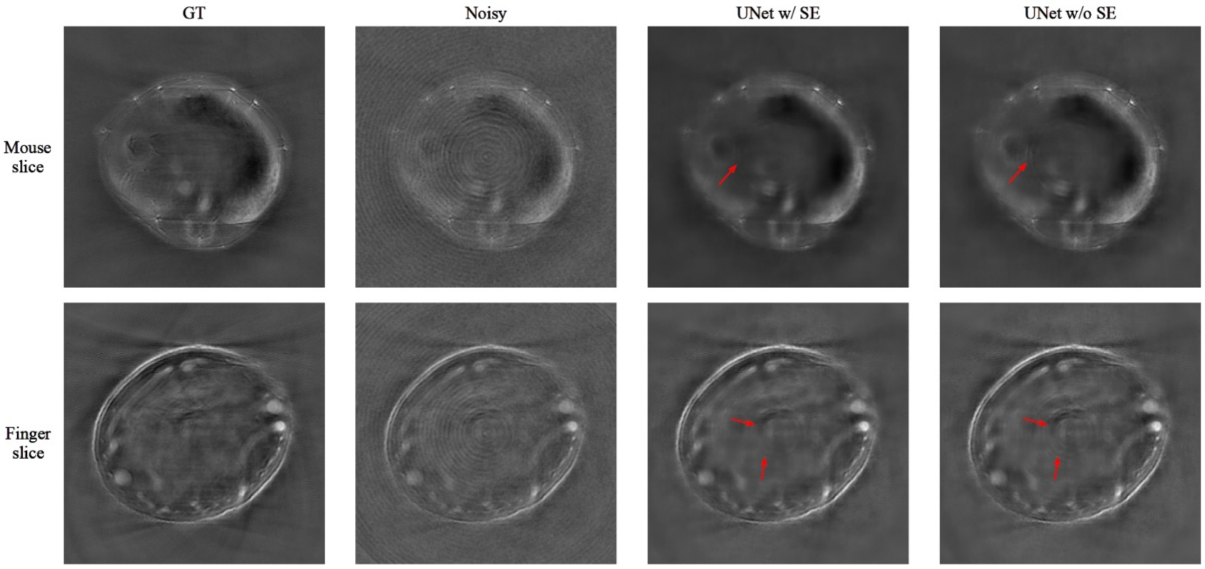

Supplementary Fig. S6

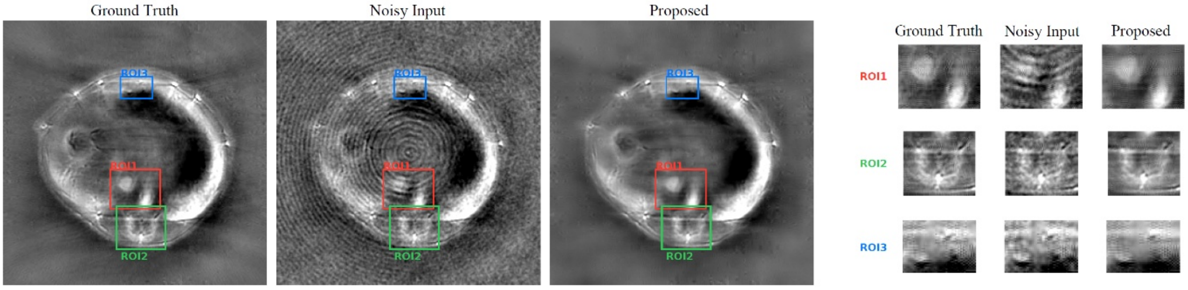

ROI2 corresponds to the spinal cord, while ROI1 contains major vessels adjacent to the spine, including the inferior vena cava and the abdominal aorta. ROI3 highlights small peripheral blood vessels, and the bright region on the right side corresponds to the liver. As illustrated, the proposed method effectively preserves the morphology and boundaries of these key structure.

Supplementary Fig. S7

Supplementary Table S1

Comparison of Noisy Input, Diffusion baseline, and the Proposed SE-UNet method using full-reference metrics against Ground Truth over 60 test images. Values are reported as mean ± standard deviation.

Method	SSIM (mean ± std)	PSNR (dB, mean ± std)	VIF (mean ± std)
Noisy Input	0.511 ± 0.143	23.757 ± 3.099	0.270 ± 0.080
Diffusion	0.688 ± 0.109	25.308 ± 3.521	0.253 ± 0.094
**Proposed**	**0.875 ± 0.057**	**29.430 ± 2.635**	**0.438 ± 0.093**

Notes:

1. The diffusion baseline follows a standard diffusion denoising formulation in which Gaussian noise is progressively added in the forward process and removed in the reverse process. The baseline was trained on the same clean SSL images under a commonly used training paradigm; it does not explicitly model a measured LED background-noise distribution, which would require paired acquisition and substantially higher sampling cost. This comparison is therefore intended to position our method against a representative modern deep-learning denoiser under a standard diffusion training setup, rather than to claim universal dominance across all diffusion variants or specialized retraining strategies.

2. Diffusion metric behavior. The diffusion baseline improves SSIM and PSNR on average, but the SSIM gain may be modest in some cases and its mean VIF can be comparable to or lower than the noisy input. This is plausible because diffusion denoisers may introduce mild prior-driven smoothing that attenuates weak edges/fine textures together with noise—sometimes limiting SSIM gains and reducing information-fidelity metrics such as VIF, especially in low-contrast ROIs. Moreover, the diffusion baseline follows a standard Gaussian diffusion paradigm and does not explicitly model measured LED background noise/structured ring-like artifacts, so a distribution mismatch can further constrain improvements in SSIM for LED-style degradations.

Supplementary Table S2.

To assess embedded-device deployability, we benchmarked the inference latency of the proposed SE-UNet on an NVIDIA Jetson Orin Nano using a TensorRT FP16 deployment with a fixed input shape. Measurements correspond to the end-to-end network forward pass at batch size 1 after engine build and warm-up. Reported latency may vary with power mode, clock settings, and deployment configuration.

Table below summarizes the benchmark configuration and measured latency.

Model	Platform	Power mode	Precision / engine	Input shape	Batch	Latency (ms/frame)
SE-UNet	NVIDIA Jetson Orin Nano	nvpmodel 15W	TensorRT FP16, static pre-built engine (fixed shape)	480 × 480	1	≈ 16 (median)

Note: Latency includes kernel-launch overhead at batch size 1 and excludes image I/O and any optional pre/post-processing. Mish activations aresupported via a composition of standard operators (Softplus and Tanh) within TensorRT.
